# Genetic Basis and Genotype–Phenotype Correlations in Han Chinese Patients with Idiopathic Dilated Cardiomyopathy

**DOI:** 10.1038/s41598-020-58984-7

**Published:** 2020-02-10

**Authors:** Xin-Lin Zhang, Jun Xie, Rong-Fang Lan, Li-Na Kang, Lian Wang, Wei Xu, Biao Xu

**Affiliations:** Department of Cardiology, Affiliated Drum Tower Hospital, Nanjing University School of Medicine, Nanjing, China

**Keywords:** Cardiology, Diseases

## Abstract

Dilated cardiomyopathy (DCM) is one of the leading causes of heart failure. A large proportion of genetic cause remains unexplained, especially in idiopathic DCM. We performed target next-generation sequencing of 102 genes which were known causes or candidate genes for cardiomyopathies and channelpathies in 118 prospectively recruited Han Chinese patients with idiopathic DCM. 41 of the 118 patients carried 40 pathogenic or likely pathogenic variants, providing a molecular diagnosis in 34.7% of patients. 32 of these variants were novel. *TTN* truncating variants were predominant, with a frequency of 31.0%, followed by variants of *LMNA* (14.3%), *RBM20* (4.8%), and *NEXN* (4.8%). These 4 genes accounted for over half variants identified. No significant difference in clinical characteristics or rates of reaching the composite end point (cardiac transplantation and death from cardiac causes) between pathogenic or likely pathogenic variant carriers and noncarriers (hazard ratio 1.11, 95% CI: 0.41 to 3.00), or between patients with *TTN* truncating variants or without (hazard ratio 0.49, 95% CI: 0.36 to 6.10). In our prospective study, we first determined the overall genetic profiles and genotype-phenotype correlations in Han Chinese idiopathic DCM patients, which could provide insight for genetic diagnosis of DCM in this population.

## Introduction

Dilated cardiomyopathy (DCM) is the one of the leading causes of heart failure and sudden death, and the most common cause of heart transplantation, affecting approximately 1 in 250 individuals^1^. DCM is a progressive disease, with 50% of patients reported to die within 5 years of diagnosis without transplantation^2^. DCM frequently has a genetic etiology, and multiple causative genes have been discovered. The genetic basis of DCM is highly diverse; over 30 genes have been identified as the potentially disease-causing genes^1,3^. About 25–30% of individuals with DCM have a familial form of the disease^1,4^. Truncating variants in *TTN*, which encodes titin, account for up to 25% of familial DCM^5^. A large proportion of genetic cause of DCM remains unexplained, especially in idiopathic DCM^6^.

Next-generation sequencing (NGS) approaches have enabled rapid genetic testing, particularly for large genes such as *TTN* which are hard to sequence with traditional methods. Using NGS, researchers have characterized the genetic atlas of DCM in Caucasian population^7,8^. Zhao and colleagues performed NGS of 25 genes in 21 Chinese patients^9^, but the number of genes and patients were limited, and the most commonly pathogenic gene in DCM—*TTN* was not included in their sequencing panel. Also, understanding the potential genotype-phenotype correlations may identify high-risk patients in this condition. In this study, we developed a custom “cardiomyopathy panel” containing 102 genes which were known causes or candidate genes for cardiomyopathies and channelpathies. We prospectively recruited 118 unrelated patients with idiopathic DCM and performed target NGS in this cohort to determine the molecular characterization of this cohort and to examine the genotype-phenotype correlations.

## Results

### Clinical characteristics

Our study consisted of 118 unrelated DCM patients of Han Chinese origin. Baseline characteristics of these patients are summarized in Table 1. Of the 118 DCM patients, 75% were male, and the mean age at diagnosis was 55.9 ± 14.7 years. The mean left ventricular ejection fraction (LVEF) was 30.2 ± 6.8%. Beta receptor blocker was used in 81% of patients, angiotensin converting enzyme inhibitor or angiotensin receptor blocker in 82% of patients, and aldosterone antagonists in 81% of patients, which indicated that most of these patients received standard therapy for heart failure. Thirty-one percent of patients received implantable cardioverter defibrillator (ICD) or cardiac resynchronization therapy with defibrillator (CRTD). Female patients had similar clinical characteristics as compared with male patients, except that the rate of smoking was lower than male patients.

**Table 1:** Patient characteristics stratified by variation status.

Characteristics	All	Variants present	Variants absent	*P* value	*TTN* variants present	*TTN* variants absent	*P* value	*LMNA* variants present	*LMNA* variants absent	*P* value
Number	118	41	77		13	105		6	112	
Male (%)	89 (75%)	29 (70.7%)	60 (77.9%)	0.50	11 (85%)	78 (74%)	0.52	6 (100%)	83 (74%)	0.33
Age of diagnosis (years)	55.9 ± 14.7	53.9 ± 14.0	56.9 ± 15.1	0.30	54.4 ± 15.6	56.1 ± 14.7	0.69	45.3 ± 15.5	56.5 ± 14.6	0.07
Smoking (%)	41 (35%)	14 (34.1%)	27 (35.1%)	1.0	6 (46%)	35 (33%)	0.37	4 (667%)	37 (33%)	0.18
Diabetes (%)	20 (17%)	9 (21.9%)	11 (14.3%)	0.32	3 (23%)	17 (16%)	0.46	2 (33%)	18 (16%)	0.31
Systolic BP (mmHg)	123.2 ± 19.9	120.4 ± 18.7	123.2 ± 20.5	0.96	119.5 ± 19.8	123.7 ± 19.9	0.49	111.2 ± 9.2	123.9 ± 20.1	0.13
Diastolic BP (mmHg)	77.3 ± 15.6	80.3 ± 14.9	75.8 ± 15.8	0.15	74.3 ± 12.8	77.6 ± 15.9	0.49	72.7 ± 9.2	77.5 ± 15.9	0.46
β receptor blocker (%)	96 (81%)	32 (78.0%)	64 (83.1%)	0.60	11 (85%)	85 (81%)	1.0	5 (83%)	91 (81%)	0.52
ACEI/ARB (%)	97 (82%)	31 (75.7%)	66 (85.7%)	0.21	10 (77%)	87 (85%)	0.70	6 (100%)	91 (81%)	1.0
Diuretic (%)	110 (92%)	32 (78.0%)	68 (88.3%)	0.18	13 (100%)	97 (92%)	0.60	6 (100%)	104 (93%)	1.0
Aldosterone antagonists (%)	95 (81%)	34 (82.9%)	61 (79.3%)	0.81	10 (77%)	85 (81%)	0.72	6 (100%)	89 (79%)	0.61
Digoxin (%)	38 (32%)	14 (34.1%)	24 (31.1%)	0.84	4 (31%)	34 (32%)	1.0	2 (33%)	36 (32%)	1.0
ICD/CRTD (%)	36 (31%)	10 (24.3%)	26 (33.8%)	0.40	5 (38%)	31 (30%)	0.53	2 (33%)	34 (30%)	1.0
LVEF (%)	30.2 ± 6.8	29.2 ± 6.3	30.7 ± 6.9	0.27	29.2 ± 7.2	30.3 ± 6.7	0.58	30.8 ± 6.4	30.2 ± 6.8	0.81
LVEDD (cm)	7.1 ± 0.9	7.08 ± 0.76	7.04 ± 0.91	0.82	6.9 ± 1.0	7.1 ± 0.9	0.44	7.2 ± 0.5	7.0 ± 0.9	0.58
IVSTD (cm)	0.9 ± 0.6	0.97 ± 0.72	0.91 ± 0.52	0.63	0.9 ± 0.1	0.9 ± 0.6	0.64	0.82 ± 0.1	0.94 ± 0.6	0.64
LAD (cm)	5.2 ± 0.9	5.02 ± 0.92	5.21 ± 0.83	0.26	5.01 ± 0.8	5.21 ± 0.8	0.33	5.86 ± 1.5	5.15 ± 0.7	0.02

Data are mean ± standard deviation, number, or percent.

ACEI, angiotensin converting enzyme inhibitor; ARB, angiotensin receptor blocker; BP, blood pressure; CRTD, cardiac resynchronization therapy (with defibrillator); DCM, dilated cardiomyopathy; DM, diabetes mellitus; ICD, Implantable cardioverter defibrillator; IVSTD, interventricular septal end-diastolic thickness; LAD, left atrial diameter; LVEDD, left ventricular end-diastolic diameter; LVEF, left ventricular ejection fraction.

### Cardiomyopathy panel coverage

We performed deep sequencing with the Cardiomyopathy panel covering the coding exons and splice junctions of 102 genes (Table S1). A total of 300 Mb of sequence was yielded per sample. The NGS captured 99.5% of the target region, and 60% of all reads are mapped to our designed target regions. A mean coverage of 281 × was reached and an average of 93.7% of target regions were covered to a depth of at least 20×. We provided the bed file for capture targets, reads covered <20 × in ~20% samples, and the percentage of patients with a <20 × depth in target regions in supplementary datasets (datasets 1 to 3).

### Landscape of genetic alterations in DCM

In total, 956 unique genetic variants were identified in 118 DCM patients. An average of 298 variants was detected in each patient in the target region with over 20 × coverage. Variants that were rare (defined here by MAF <0.01% in the ExAC and gnomAD databases) and altered protein sequences (truncating, missense, in-frame insertions/deletions [indels]) in a set of 102 cardiomyopathy-associated genes were evaluated. After filtering, a total of 65 different rare variants in 28 genes were found. Among these 65 rare variants, 43 were missense variants, 6 were nonsense variants, 14 were frameshift indels, and 2 were in-frame indels (Table S2). Of the 118 patients’ samples, 59 (50%) harbored at least one rare variant. Eighteen of the 43 (41.8%) rare missense variants were predicted to be “damaging” according to various prediction programs. Specially, the Combined Annotation Dependent Depletion (CADD) scores of all these 18 variants were higher than 20, indicating rarity and deleteriousness of these variants. Altogether, 40 variants were considered as pathogenic or likely pathogenic in our study (Table 2 and Table 3), 8 of which were recorded in HGMD or the ClinVar database and/or supported by published data and the remaining 32 were novel. All pathogenic or likely pathogenic variants were heterozygous, and most of these variations were found in our study are private, except 2 variants (*LMNA*: c.568 C > T p.R190W; *RBM20*: c.2017C > T p.R673W). Each of these LMNA and RBM20 variants were shared in 2 unrelated DCM patients and published in other populations^10,11^.

**Table 2:** Pathogenic and likely pathogenic truncating variants or in-frame insertions/deletions in Chinese DCM cohort (22 variants).

Gene	Transcript	Exon	Nucleotide Change	Amino Acid Change	Effect	Publication	MAF gnomAD	MAF ExAC	*TTN* band
*ADRB1*	NM_000684	1	c.763_764delGT	p.255_255delV	Frameshift	—	0	0	—
*ANK2*	NM_001148	38	c.5772_5773insAAAAC	p.K1924fs	Frameshift	—	0	0	—
*CBL*	NM_005188	9	c.1363_1364insATG	p.Y455delinsYD	Nonframeshift	—	0	0	—
*EMD*	NM_000117	6	c.596 C > G	p.S199X	Nonsense	—	0	0	—
*LMNA*	NM_170707	8	c.1477 C > T	p.Q493X	Nonsense	—	0	0	—
*LMNA*	NM_170707	9	c.1590delC.	p.L530fs	Frameshift	—	0	0	—
*MYBPC3*	NM_000256	24	c.2541 C > G	p.Y847X	Nonsense	Yes	0	0	—
*NEXN*	NM_144573	12	c.1587_1589delAAG	p.529_530del	Nonframeshift	—	0	0	—
*TTN*	NM_001267550	352	c.98650_98651insT	p.S32884fs	Frameshift	Yes	0	0	A band
*TTN*	NM_001267550	258	c.48325_48326insT	p.L16112fs	Frameshift	—	0	0	A band
*TTN*	NM_001267550	326	c.78749 T > A	p.L26250X	Nonsense	—	0	0	A band
*TTN*	NM_001267550	335	c.89855delT	p.L29952fs	Frameshift	—	0	0	A band
*TTN*	NM_001267550	358	c.101000_101001delAT	p.Y33667fs	Frameshift	—	0	0	A band
*TTN*	NM_001267550	342	c.94931delA	p.E31644fs	Frameshift	—	0	0	A band
*TTN*	NM_001267550	274	c.52154 C > A	p.S17385X	Nonsense	—	0	0	A band
*TTN*	NM_001267550	248	c.46051delA	p.R15350fs	Frameshift	—	0	0	I band
*TTN*	NM_001267550	49	c.14251delT	p.S4751fs	Frameshift	—	0	0	I band
*TTN*	NM_001267550	255	c.47843_47844insT	p.I15948fs	Frameshift	—	0	0	A band
*TTN*	NM_001267550	246	c.45550 C > T	p.Q15184X	Nonsense	—	0	0	I band
*TTN*	NM_001267550	226	c.41377delG	p.V13793fs	Frameshift	—	0	0	I band
*TTN*	NM_001267550	326	c.71024_71027del	p.23675_23676del	Frameshift	—	0	0	A band
*VCL*	NM_003373	6	c.632delT	p.I211fs	Frameshift	—	0	0	—

**Table 3:** Pathogenic and likely pathogenic missense variants in Chinese DCM cohort (18 variants).

Gene	Transcript	Exon	Nucleotide Change	Amino Acid Change	RsID	Effect	Publication	MAF gnomAD	MAF ExAC	SIFT score	PolyPhen2 HDIV score	MutationTaster score	CADD score
*ANKRD1*	NM_014391	7	c.682 A > G	p.R228G	—	Missense	—	0	0	0.001	0.989	1	26.6
*CACNA1C*	NM_000719	46	c.6035 G > A	p.R2012Q	rs772606843	Missense	Yes	—	9.96e-06	0.048	1	1	24.3
*DES*	NM_001927	4	c.887 A > G	p.Y296C	—	Missense	—	0	0	0	1	0.999	27.5
*DMD*	NM_004010	59	c.8480 T > A	p.L2827Q	—	Missense	—	0	0	0.001	1	1	26
*DSG2*	NM_001943	15	c.2959 G > T	p.V987F	rs141405267	Missense	—	6.46e-05	7.59e-05	0.041	0.986	1	23.8
*LMNA*	NM_170707	3	c.568 C > T	p.R190W	rs59026483	Missense	Yes	0	0	0	1	1	35
*LMNA*	NM_170707	6	c.1088 T > C	p.L363P		Missense	Yes	0	0	0	1	1	28.7
*LMNA*	NM_170707	10	c.1633C > A	p.R545S		Missense	—	0	0	0.016	0.995	1	24.2
*MYH6*	NM_002471	36	c.5539 C > T	p.R1847W	rs752718246	Missense	—	8.165e-06	6.599e-05	0	1	1	34
*MYH7*	NM_000257	25	c.3134 G > A	p.R1045H	rs397516178	Missense	Yes	0	3.295e-05	0	1	1	28.7
*MYPN*	NM_032578	2	c.468 C > G	p.D156E	—	Missense	—	6.456e-05	0	0.012	1	1	24.5
*NEXN*	NM_144573	8	c.835 C > A	p.R279S	rs146245480	Missense	—	0	2.512e-05	0.007	0.987	1	29.6
*PKP2*	NM_001005242	1	c.125 G > A	p.G42E	rs748880850	Missense	—	0	0	0.016	1	0.962	29.4
*PRDM16*	NM_022114	7	c.1006 C > T	p.R336C	rs748880850	Missense	—	2.514e-05	6.466e-05	0.019	1	1	34
*RYR2*	NM_001035	18	c.1748C > A	p.P583Q	—	Missense	—	0	0	0.001	0.98	1	26.4
*RBM20*	NM_001134363	9	c.2017C > T	p.R673W	rs397516599	Missense	Yes	9.699e-05	5.395e-05	0	1	0.998	28.9
*SCN5A*	NM_001160161	25	c.4357 C > A	p.Q1453K	—	Missense	—	—	—	0.001	0.996	0.979	23.7
*TNNT2*	NM_000364	4	c.472 C > T	p.R158W	rs730881123	Missense	Yes	0	0	0	1	1	35

**Figure 1:**
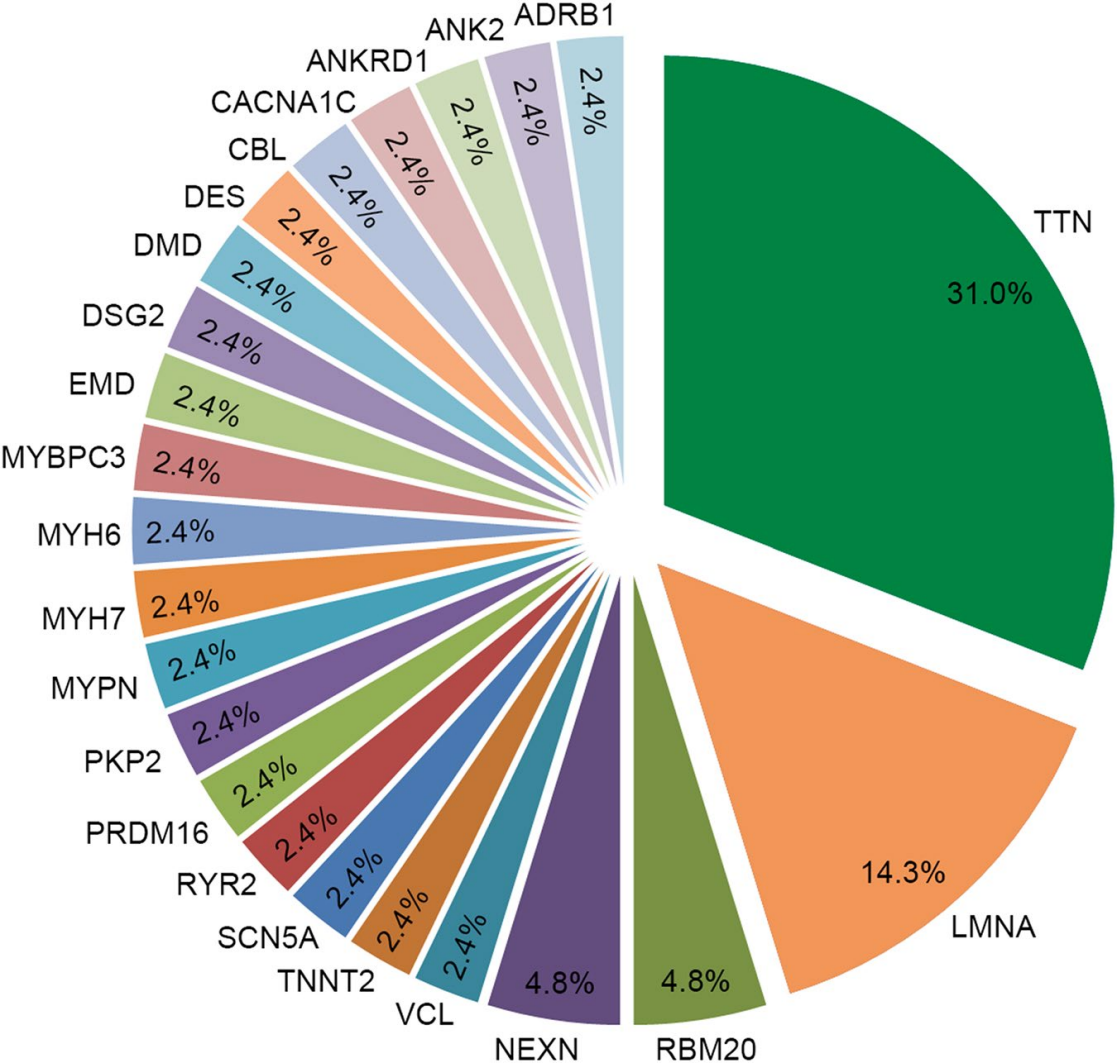
The distribution of pathogenic or likely pathogenic variants identified in the idiopathic dilated cardiomyopathy cohort.

In our cohort, 41 out of the 118 patients (34.7%) carried pathogenic or likely pathogenic variants. The distribution of these pathogenic or likely pathogenic variants was not equal among genes, as was presented in Table 2 and Table 3. *TTN* truncating variants were predominant, with a frequency of 31.0%, followed by variants of *LMNA* (14.3%), *RBM20* (4.8%), and *NEXN* (4.8%). Other pathogenic or likely pathogenic variants present at low frequency (2.4%) in the study population were identified in *ADRB1*, *ANK2*, *ANKRD1*, *CACNA1C*, *CBL*, *DES*, *DSG2*, *DMD*, *EMD*, *MYBPC3*, *MYH6*, *MYH7*, *MYPN*, *PKP2*, *PRDM16*, *RYR2*, *SCN5A*, *TNNT2* and *VCL*, each with 1 variant (Fig. 1). *TTN* truncating variants were observed in 13 of the 118 patients (11.0%). As expected, *TTN* truncating variants were nonrandomly distributed within titin^5^, with most variants located in the titin A-band region and others in I-band region (Table 2). All these *TTN* truncating variants are expressed in both the N2BA and N2B isoforms and constitutively expressed in the heart^12^. No patient carried multiple pathogenic or likely pathogenic variants.

### Genotype–phenotype correlations

**Figure 2:**
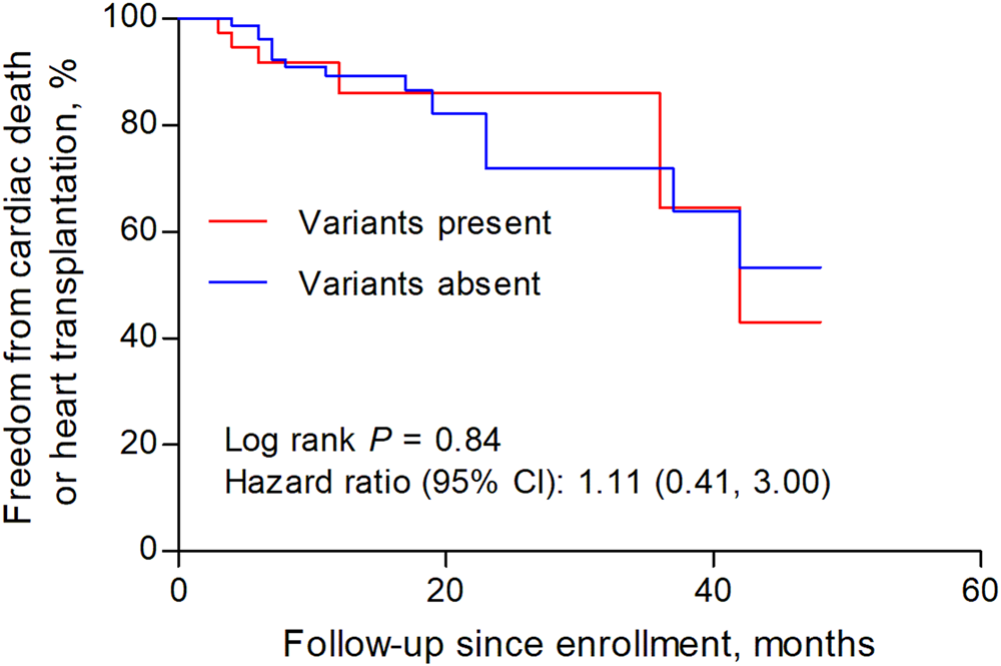
Survival curves comparing freedom from the composite endpoint of cardiac death and heart transplantation in patients with and without pathogenic or likely pathogenic variants.

**Figure 3:**
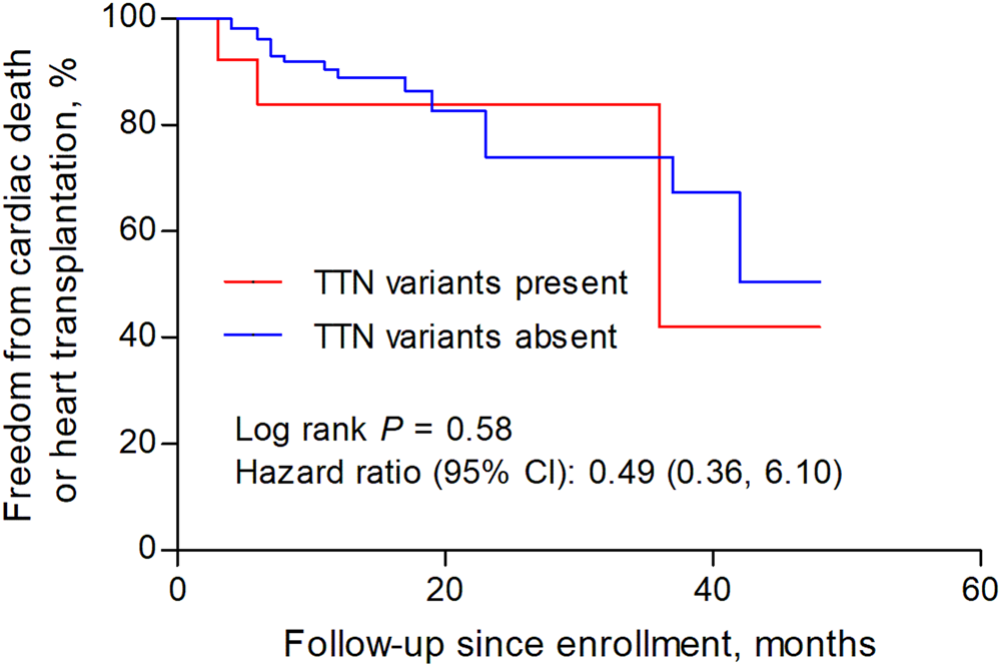
Survival curves comparing freedom from the composite endpoint of cardiac death and heart transplantation in patients with and without rare *TTN* truncating variants.

**Figure 4:**
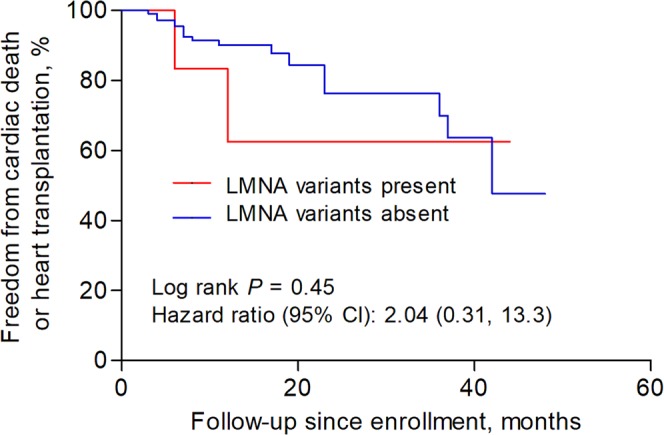
Survival curves comparing freedom from the composite endpoint of cardiac death and heart transplantation in patients with and without pathogenic or likely pathogenic *LMNA* variants.

We compared the clinical characteristics of patients with and without pathogenic or likely pathogenic variants. As shown in Table 1, the age of diagnosis was similar between patients with or without variants. There were no significant differences in sex, treatment and dosage of common medical therapy for heart failure among these 2 groups. Also, patients present or absent with these variants had similar left ventricular ejection fractions, left ventricular end-diastolic diameter and other echocardiography parameters. In terms of the composite endpoint of cardiac death and heart transplantation, there was no significant difference between patients with and without pathogenic or likely pathogenic variants (hazard ratio 1.11, 95% confidence interval 0.41 to 3.00, *P* = 0.84, Fig. 2). We also made similar comparisons between patients who tested positive or negative with *TTN* truncating or *LMNA* variants in our study. No significant differences in clinical characteristics (Table 1) or follow-up endpoints were detected between DCM patients present with *TTN* truncating variants and those absent (hazard ratio 0.49, 95% confidence interval 0.36 to 6.10, *P* = 0.58, Fig. 3). *LMNA* genotype-positive subjects seem to have a younger age of diagnosis of DCM (45.3 ± 15.5 vs. 56.5 ± 14.6; *P* = 0.07) and larger left atrial (5.86 ± 1.5 vs. 5.15 ± 0.7; *P* = 0.02) than those negative patients. The outcome difference between these 2 groups was not significant and had wide confidence intervals (hazard ratio 2.04, 95% confidence interval 0.31 to 13.3, *P* = 0.45, Fig. 4). We did not compare clinical characteristics and outcomes between other individual gene variants because of limited number of patients with an individual variant in these genes.

## Discussion

We developed and utilized a high-quality 102-gene targeted sequencing panel and sequenced 118 idiopathic DCM patients. To the best of our knowledge, this is the one of the very few genetic studies on idiopathic DCM with a prospective design. For the first time we revealed the distribution of disease-causing genes and the pathogenic or likely pathogenic variants of DCM patients in Chinese population. In our prospective cohort, 41of the 118 patients carried 40 pathogenic or likely pathogenic rare variants, with *TTN*, *LMNA*, *RBM20* and *NEXN* being the 4 most frequently affected genes, accounting for over half these variants. We also revealed that no significant difference in baseline clinical characteristics or rates of reaching the composite end point (cardiac transplantation and death from cardiac causes) between pathogenic or likely pathogenic variant carriers and noncarriers, or between patients with *TTN* truncating variants and without. *LMNA* genotype-positive subjects seem to have a younger age of diagnosis of DCM and larger left atrial than those negative patients, but the outcome difference was not significant.

Our study showed that about one third DCM patients carried pathogenic or likely pathogenic variants, a frequency similar to those reported in in Japanese and Finish populations^8,13^. *TTN* truncating variants remain the most common variants in our Chinese cohort. The observed frequency of *TTN* truncating variants among our idiopathic DCM population (11.0%) lie around the lower limits of previously published cohorts, which reported a frequency between 12% and 27% mainly in Caucasian population^5,7,8,14^. As *TTN* truncating variant frequency varied with the disease severity of DCM, with a higher frequency in patients with severe, end-stage or clearly familial cases of DCM^5^, it is plausible that the relatively low frequency in our cohort was due to the exclusion of familial DCM and the inclusion of all DCM patients in hospital and the outpatient clinic in our analysis. Ethnic difference cannot be simply attributed to and needs further investigation, as no correlation between ethics and frequency of *TTN* truncating variants has been noted: a Caucasian population reported a frequency of 12.0%^14^, and a Japanese study reported 16.7%^13^.

Truncating *TTN* variants are not infrequent in the general population. It is estimated that the prevalence of Truncating *TTN* variants in the general population is ~0.4%^15^. Therefore, how to interpret truncating *TTN* variants is an open question. The etiological fraction for *TTN* truncating variants in DCM patients was ~97% when limiting to variants in exons that are constitutively expressed in the heart^15,16^, higher than the causative cut-off value of 0.9 or 0.95 recommended by consensus guidelines for variant interpretation in genetic testing^17^. All truncating variants in our DCM cohort lied in the constitutive exons, and therefore it is reasonable to classify them as pathogenic or likely pathogenic. Meanwhile, although most individuals with *TTN* truncating variants may not develop DCM over time, these variants are not necessarily phenotypically silent. In fact, there are studies showing morphological and functional abnormity in individuals in the general population with *TTN* truncating variants^16^.

Our study did not reveal a significant association between *TTN* truncating variants and the prognosis. Patients with *TTN* truncating variants had similar cardiac phenotypes and a similar risk for the clinical endpoint of cardiac transplantation and death from cardiac causes. This observation was in line with that reported by Tayal and colleagues, which showed a similar prognosis for DCM patients with *TTN* truncating variants, reaching the primary composite end point comprising cardiovascular mortality, major arrhythmic events, and major heart failure events^14^. Although the same study group showed an increased propensity to arrhythmia early in the disease course, the long-term arrhythmic events on follow-up proved similar^14,18^. Akinrinade and colleagues concluded in the Finnish population that adverse outcomes in patients with *TTN* truncating variants were indistinct from those other gene variant groups except *LMNA* variants^8^. In several other studies, however, patients with *TTN* truncating variants were reported to be less severe at presentation and to be associated with a favorable response to treatment than patients with *LMNA* variants or patients negative for *TTN* and *LMNA* genes^13,19^. Altogether, the published data on genotype-phenotype associations in DCM cannot provide a clear correlation between *TTN* truncating variants and clinical phenotype. Whether *TTN* truncating variants could predict clinical outcomes in the longer-term remain to be established in larger studies^15^. By contrast, accumulating evidence proved that DCM associated with *LMNA* variants had a higher risk for sudden cardiac death (SCD), cardiac transplantation, cardiac conduction disturbance, and atrial or ventricular arrhythmias^20–22^, indicating their potential role in risk stratification^23^. With limited power, our analysis did not show a genotype–phenotype correlation between *LMNA* variants and risk for the primary outcome.

Our study has several limitations. This study population is from a single, highly advanced center, patients may have been subjected to a selection bias. Second, we did not include familial DCM in our analysis and thus could not represent the general DCM cohort. Third, the genotype–phenotype findings should be interpreted with caution due to the limited statistical power and thus be considered as hypotheses generating; a larger number of cohorts are needed to establish these genotype–phenotype associations. Fourth, several newly discovered cardiomyopathy-related genes, including *FLNC*^24^ and *FBXO32*^25^, were not included in the study panel due to description of pathogenicity after the design of the study.

## Conclusions

In our prospective idiopathic DCM cohort, about one third patients carried pathogenic or likely pathogenic variants, with *TTN*, *LMNA*, *RBM20* and *NEXN* accounting for over half these variants. There is no significant difference in baseline clinical characteristics or rates of reaching the composite end point between pathogenic variant carriers and noncarriers, or between patients with *TTN* truanting variants or without.

## Materials and Methods

### Subjects

This study was conducted in accordance with the ethical guidelines of the Declaration of Helsinki. The Institutional Review boards at Nanjing University approved the study, and written informed consent was obtained from all participants or their legal representatives. The study population comprised 118 unrelated patients with idiopathic DCM. All patients were of Han Chinese origin, and were prospectively recruited to the affiliated Drum Tower Hospital, Nanjing University School of Medicine between 2011 and 2015.

### Diagnosis of DCM

DCM was diagnosed according to the ESC (European Society of Cardiology) criteria^26^. Briefly, all patients had to have a reduced systolic function of the left ventricle (LVEF <45%) and a dilated left ventricle (left ventricular end-diastolic dimension >117% of the predicted value corrected for body surface area and age). Other identifiable causes such as hypertensive heart disease, primary valve disease, congenital heart disease, excess alcohol consumption and significant coronary artery disease were excluded. All recruited patients with DCM underwent family screening. Familial DCM was defined if at least 1 additional family member was diagnosed with DCM or encountered sudden cardiac death (up to third-degree relative). We did not include patients with familial DCM in our analyses.

### Clinical data

Baseline demographic and clinical information was obtained from each subject during the index visit, through medical history interview, physical examination, electrocardiogram (ECG), and transthoracic echocardiography. Whole blood was collected for further genetic analysis. Follow-up data was collected from hospital care records and patient questionnaires by physicians blinded to the genetic data. The primary end point in this analysis was a composite of cardiac death and heart transplantation during the follow-up period.

### Echocardiography

An experienced operator who was blinded of the genotype and clinical status of study subject performed the echocardiography test. We used a Philips Sonos 5500 ultrasound system to obtain the M-mode and 2-dimensional images, and acquire the Doppler recordings. We used the Simpson biplane method to calculate LVEF. We measured the left ventricular end-diastolic diameter and left ventricular septal and posterior wall thickness from 2-dimensional images.

### Targeted next-generation sequencing

Genomic DNA was extracted from whole blood using the QIAamp DNA Blood Mini Kit (Qiagen). A panel consisting of 102 genes which were known causes or candidate genes for cardiomyopathies and channelpathies (Table S1) was designed. Targeted next-generation sequencing, which included library construction, capture, and sequencing, was carried out. Targeted gene enrichment was performed with the GenCap Custom Enrichment Kit according to the GenCap protocol, as described previously^27–29^. Captured DNA libraries were sequenced with the Illumina HiSeq. 2000 instrument (Illumina, San Diego, CA), producing 100-bp paired-end reads.

### Variant classification

Mapping of the sequencing reads to the human genome reference sequence (hg19) was performed with the burrows-wheeler alignment tool (BWA, http://bio-bwa.sourceforge.net/)^30^. The Short Oligonucleotide Analysis Package (SOAPsnp) and the Genome Analysis Toolkit (GATK, https://www.broadinstitute.org/gatk/) were used to discover single-nucleotide polymorphism (SNP) and insertion-deletion (indel), respectively^31,32^. Gene related annotation was mainly done with ANNOVAR (http://wannovar.wglab.org/). Coding-sequence variants met quality metrics for mapping, read depth, and allelic balance were evaluated. The pathogenicity of a variant was determined based on frequency in the population and in silico prediction. We excluded synonymous variants, intronic variants outside of the flanking regions, and variants with a minor allele frequency greater than 0.01% in the National Heart, Lung, and Blood Institute ESP (Exome Sequencing Project), the 1000 Genomes database (http://browser.1000genomes.org), the dbSNP137 database (http://www.ncbi.nlm.nih.gov/snp), Exome Aggregation Consortium Browser (http://exac.broadinstitute.org/), the Genome Aggregation (gnomAD) databases (http://gnomad.broadinstitute.org/), and a cohort of 500 in-house whole-exome controls. All missense variants were subjected to in silico analysis with functional annotation algorithms including SIFT (http://sift.jcvi.org/), PolyPhen2 (http://genetics.bwh.harvard.edu/pph2/), GERP +  + (http://mendel.stanford.edu/sidowlab/downloads/gerp/index.html), and MutationTaster (http://www.mutationtaster.org/). In addition, CADD scores were obtained to assess missense variant pathogenicity (https://cadd.gs.washington.edu/score). Variants were checked for known pathogenic relationships with cardiovascular diseases in the Human Gene Mutation Database (HGMD, http://www.hgmd.org). Rare coding-sequence variants resulting in premature truncation (frameshift insertions/deletions, stop gain, splice donor or acceptor site gain or loss) and rare missense variants declared to be disease-causing by analytical algorithms were considered as pathogenic or likely pathogenic in our study^33^. *TTN* missense variants were not considered likely pathogenic because they are common and present a challenge for bioinformatic classification, especially when informative families are not available^34^.

### Statistical analysis

Variables were presented as number (percentage) or mean ± standard deviation, as appropriate. Categorical variables were compared using chi-square test or Fisher exact test, while continuous variables with independent sample’s t-test. The Kaplan-Meier curves were constructed, and differences between survival curves were compared with log-rank test. A two-sided *p*-value < 0.05 was considered significant. All statistical analyses were performed with SPSS version 17.0.

## Supplementary information

Supplementary materials.

Dataset 1.

Dataset 2.

Dataset 3.

## Data Availability

All materials were available in the manuscript and supplementary materials.
