# Supplementary material for: Genetic Basis and Genotype–Phenotype Correlations in Han Chinese Patients with Idiopathic Dilated Cardiomyopathy
Source: Sci Rep. 2020 Feb 10;10:2226. doi: 10.1038/s41598-020-58984-7 (PMC7010767; doi:10.1038/s41598-020-58984-7)
Supplement: Supplementary file 1 — Supplementary materials. [file 41598_2020_58984_MOESM1_ESM.docx]

**Supplementary Materials**

**Genetic Basis and Genotype–Phenotype Correlations in Han Chinese Patients with Idiopathic Dilated Cardiomyopathy**

Xin-Lin Zhang, Jun Xie, Rong-Fang Lan, Li-Na Kang, Lian Wang, Wei Xu, Biao Xu

**Table S1.** List of genes included in the targeted next-generation sequencing.

| *AARS2* | *CALR3* | *DTNA* | *KCNE1* | *LDB3* | *MYPN* | *RBM20* | *SNTA1* | *TNNT2* |
| --- | --- | --- | --- | --- | --- | --- | --- | --- |
| *ABCC9* | *CASQ2* | *EMD* | *KCNE2* | *LMNA* | *NEXN* | *RYR2* | *SOS1* | *TNNT3* |
| *ACTC1* | *CAV3* | *EYA4* | *KCNE3* | *LAMA4* | *NOS3* | *SAGD* | *TAZ* | *TPM1* |
| *ACTN2* | *CBL* | *FHOD3* | *KCND3* | *MIB1* | *NRAS* | *SCN1B* | *TCAP* | *TTN* |
| *AKAP9* | *CRYAB* | *FKTN* | *KCNH2* | *MLP* | *OBSCN* | *SCN3B* | *TGFB1* | *TTR* |
| *ANK2* | *CSRP3* | *GATAD1* | *KCNIP2* | *MYBPC3* | *PDLIM3* | *SCN4B* | *TGFB3* | *VCL* |
| *ANKRD1* | *DES* | *GLA* | *KCNJ2* | *MYH6* | *PKP2* | *SCN5A* | *TMEM43* |  |
| *BAG3* | *DMD* | *GPD1L* | *KCNJ5* | *MYH7* | *PLN* | *SCO2* | *TMEM70* |  |
| *BRAF* | *DSC2* | *HCN4* | *KCNJ11* | *MYL2* | *PRDM16* | *SERCA* | *TMPO* |  |
| *CACNA1C* | *DSG2* | *ILK* | *KCNQ1* | *MYL3* | *PRKAG2* | *SGCD* | *TNNC1* |  |
| *CACNB2* | *DSG3* | *JPH2* | *KRAS* | *MYLK2* | *PTPN11* | *SHOC2* | *TNNC2* |  |
| *CACNA2D1* | *DSP* | *JUP* | *LAMP2* | *MYOZ2* | *RAF1* | *SLC25A4* | *TNNI3* |  |

**Table S2.** List of all rare variants detected in Chinese DCM cohort (65 rare variants)

| Gene | Transcript | Exon | Nucleotide Change | Amino Acid Change | RsID | Effect | Publication | MAF  gnomAD | MAF ExAC | SIFT score | PolyPhen2 HDIV score | MutationTaster score | CADD score |
| --- | --- | --- | --- | --- | --- | --- | --- | --- | --- | --- | --- | --- | --- |
| *ACTN2* | NM_001278343 | 12 | c.1303A>T | p.T435S | - | Missense | - | - | - | 1 | 0.066 | 1 | 2.402 |
| *ADRB1* | NM_000684 | 1 | c.763_764del | p.255_255del | - | Frameshift | - | - | - | - | - | - | - |
| *ANK2* | NM_001148 | 38 | c.5772_5773insAAAAC | p.K1924fs | - | Frameshift | - | - | - | - | - | - | - |
| *ANKRD1* | NM_014391 | 7 | c.682A>G | p.R228G | - | Missense | - | - | - | 0.001 | 0.989 | 1 | 26.6 |
| *BAG3* | NM_004281 | 4 | c.1028G>T | p.R343L | rs774085746 | Missense | - | - | 8.24E-06 | 0.553 | 0.001 | 0.998 | 14.41 |
| *CACNA1C* | NM_000719 | 9 | c.1359C>G | p.D453E | - | Missense | - | - | - | 0.96 | 0.999 | 0.994 | 11.58 |
| *CACNA1C* | NM_000719 | 18 | c.2486A>G | p.N829S | rs773015884 | Missense | - | 9.69E-05 | 1.24E-05 | 0.425 | 0.296 | 0.683 | 11.7 |
| *CACNA1C* | NM_000719 | 46 | c.6035G>A | p.R2012Q | rs772606843 | Missense | Yes | - | 9.96E-06 | 0.048 | 1 | 1 | 24.3 |
| *CBL* | NM_005188 | 9 | c.1363_1364insATG | p.Y455delinsYD | - | Nonfs | 0 | 0 | - | - | - | - | - |
| *CBL* | NM_005188 | 10 | c.1459A>G | p.M487V | rs17848896 | Missense | - | 9.69E-05 | 0.0001 | 0.44 | 0 | 1 | 0.06 |
| *DES* | NM_001927 | 1 | c.65C>G | p.P22R | rs748158450 | Missense | - | - | 3.92E-05 | 0.034 | 0.653 | 1 | 11.98 |
| *DES* | NM_001927 | 4 | c.887A>G | p.Y296C | - | Missense | - | - | - | 0 | 1 | 0.999 | 27.5 |
| *DMD* | NM_004010 | 59 | c.8480T>A | p.L2827Q | - | Missense | - | - | - | 0.001 | 1 | 1 | 26 |
| *DMD* | NM_004010 | 44 | c.5959C>A | p.H1987N | - | Missense | - | - | - | 0.368 | 0.991 | 0.747 | 20.3 |
| *DMD* | NM_004010 | 38 | c.5073A>T | p.K1691N | - | Missense | - | - | - | 0.258 | 0.995 | 0.784 | 22.9 |
| *DSG2* | NM_001943 | 15 | c.2959G>T | p.V987F | rs141405267 | Missense | - | 6.46E-05 | 7.59E-05 | 0.041 | 0.986 | 1 | 23.8 |
| *DSP* | NM_004415 | 23 | c.3221C>T | p.A1074V | rs745849763 | Missense | - | 3.23E-05 | 8.31E-06 | 0.097 | 0.342 | 0.789 | 23.3 |
| *EMD* | NM_000117 | 6 | c.596C>G | p.S199X | - | Nonsense | - | - | - | - | - | 1 | 35 |
| *LMNA* | NM_170707 | 3 | c.568C>T | p.R190W | rs59026483 | Missense | Yes | - | - | 0 | 1 | 1 | 35 |
| *LMNA* | NM_170707 | 6 | c.1052G>A | p.R351K | rs779749639 | Missense | - | - | 8.28E-06 | 0.536 | 0.106 | 1 | 19.93 |
| *LMNA* | NM_170707 | 6 | c.1088T>C | p.L363P | - | Missense | Yes | - | - | 0 | 1 | 1 | 28.7 |
| *LMNA* | NM_170707 | 8 | c.1477C>T | p.Q493X | rs56699480 | Nonsense | Yes | - | - | - | - | 1 | 42 |
| *LMNA* | NM_170707 | 9 | c.1590delC. | p.L530fs | - | Frameshift | - | - | - | - | - | - | - |
| *LMNA* | NM_170707 |  | c.1633C>A | p.R545S | - | Missense | - | - | - | 0.016 | 0.995 | 1 | 24.2 |
| *MYBPC3* | NM_000256 | 24 | c.2541C>G | p.Y847X | rs397515974 | Nonsense | Yes | - | - | - | - | 1 | 37 |
| *MYBPC3* | NM_000256 | 12 | c.1074T>A | p.D358E | rs775464343 | Missense | - | - | 1.67E-05 | 1 | 0 | 0.987 | 0.594 |
| *MYH6* | NM_002471 | 13 | c.1336G>A | p.A446T | rs556536964 | Missense | - | 6.47E-05 | 0.0001 | 0.019 | 0.002 | 0.801 | 22.4 |
| *MYH6* | NM_002471 | 11 | c.904C>A | p.L302M | - | Missense | - | - | - | 0.009 | 0.016 | 1 | 22.7 |
| *MYH6* | NM_002471 | 5 | c.449C>G | p.A150G | - | Missense | - | - | - | 0.001 | 0.03 | 1 | 22.5 |
| *MYH6* | NM_002471 | 5 | c.404A>G | p.N135S | rs763885051 | Missense | - | 3.23E-05 | 2.47E-05 | 0.064 | 0.005 | 0.746 | 10.77 |
| *MYH6* | NM_002471 | 3 | c.101G>A | p.R34H | rs762303505 | Missense | - | - | 8.24E-06 | 0 | 0.566 | 0.997 | 24.5 |
| *MYH7* | NM_000257 | 25 | c.3134G>A | p.R1045H | rs397516178 | Missense | Yes | - | 3.3E-05 | 0 | 1 | 1 | 28.7 |
| *MYH7* | NM_000257 | 5 | c.449C>G | p.A150G | - | Missense | - | - | - | 0.001 | 0.062 | 1 | 24.5 |
| *MYLK2* | NM_033118 | 3 | c.464T>C | p.I155T | - | Missense | - | - | - | 0.38 | 0.001 | 1 | 0.004 |
| *MYPN* | NM_032578 | 2 | c.468C>G | p.D156E | - | Missense | - | 6.46E-05 | - | 0.012 | 1 | 1 | 24.5 |
| *NEXN* | NM_001172309 | 11 | c.1395_1397delAAG | p.465_466delQRinsQ | rs754350672 | Nonfs | - | - | 1.67E-05 | - | - | - | - |
| *PKP2* | NM_001005242 | 1 | c.125G>A | p.G42E | - | Missense | - | - | - | 0.016 | 1 | 0.962 | 29.4 |
| *PRDM16* | NM_022114 | 7 | c.1006C>T | p.R336C | rs748880850 | Missense | - | 2.514e-05 | 6.466e-05 | 0.019 | 1 | 1 | 34 |
| *RBM20* | NM_001134363 | 9 | c.2017C>T | p.R673W | rs397516599 | Missense | Yes | 9.699e-05 | 5.395e-05 | 0 | 1 | 0.998 | 28.9 |
| *RYR2* | NM_001035 | 18 | c.1748C>A | p.P583Q | - | Missense | - | - | - | 0.001 | 0.98 | 1 | 26.4 |
| *SCN5A* | NM_001160161 | 25 | c.4357C>A | p.Q1453K | - | Missense | - | - | - | 0.001 | 0.996 | 0.979 | 23.7 |
| *SCO2* | NM_005138 | 2 | c.274G>A | p.E92K | - | Missense | - | - | - | 0.363 | 0.083 | 0.995 | 13.92 |
| *TNNT2* | NM_000364 | 4 | c.472C>T | p.R158W | rs730881123 | Missense | Yes | - | - | 0 | 1 | 1 | 35 |
| *TTN* | NM_001267550 | 352 | c.98650_98651insT | p.S32884fs | - | Frameshift | - | - | - | - | - | - | - |
| *TTN* | NM_001267550 | 258 | c.48325_48326insT | p.L16112fs | - | Frameshift | - | - |  | - | - | - | - |
| *TTN* | NM_001267550 | 326 | c.78749T>A | p.L26250X | - | Nonsense | - | - |  | - | - | 1 | 65 |
| *TTN* | NM_001267550 | 335 | c.89855delT | p.L29952fs | - | Frameshift | - | - |  | - | - | - | - |
| *TTN* | NM_001267550 | 358 | c.101000_101001delAT | p.Y33667fs | - | Frameshift | - | - |  | - | - | - | - |
| *TTN* | NM_001267550 | 342 | c.94931delA | p.E31644fs | - | Frameshift | - | - |  | - | - | - | - |
| *TTN* | NM_001267550 | 274 | c.52154C>A | p.S17385X | - | Nonsense | - | - |  | - | - | 1 | 62 |
| *TTN* | NM_001267550 | 248 | c.46051delA | p.R15350fs | - | Frameshift | - | - |  | - | - | - | - |
| *TTN* | NM_001267550 | 49 | c.14251delT | p.S4751fs | - | Frameshift | - | - |  | - | - | - | - |
| *TTN* | NM_001267550 | 255 | c.47843_47844insT | p.I15948fs | - | Frameshift | - | - |  | - | - | - | - |
| *TTN* | NM_001267550 | 246 | c.45550C>T | p.Q15184X | - | Nonsense | - | - |  | - | - | 1 | 61 |
| *TTN* | NM_001267550 | 226 | c.41377delG | p.V13793fs | - | Frameshift | - | - |  | - | - | - | - |
| *TTN* | NM_001267550 | 326 | c.71024_71027del | p.K23675fs | - | Frameshift | - | - |  | - | - | - | - |
| *TTN* | NM_001267550 | 360 | c.106708G>A | p.V35570I | - | Missense | - | - |  | 0.151 | 0 | 1 | 8.852 |
| *TTN* | NM_001267550 | 297 | c.58295G>A | p.R19432H | rs745631866 | Missense | - | - |  | 0.196 | 0.956 | 0.995 | 23.9 |
| *TTN* | NM_001267550 | 295 | c.57832A>G | p.I19278V | rs56025724 | Missense | - | - |  | 0.36 | 0.985 | 1 | 20.2 |
| *TTN* | NM_001267550 | 272 | c.51704G>A | p.R17235Q | rs573695008 | Missense | - | 6.48E-05 |  | 0.092 | 0.76 | 0.988 | 23.4 |
| *TTN* | NM_001267550 | 242 | c.44588C>T | p.T14863M | rs759406486 | Missense | - | 9.72E-05 |  | 0.004 | 1 | 1 | 22.7 |
| *TTN* | NM_001267550 | 101 | c.29078C>A | p.A9693E | rs767793508 | Missense | - | - |  | 0.262 | 0.004 | 1 | 22.9 |
| *TTN* | NM_001267550 | 95 | c.27583T>C | p.C9195R | rs762899501 | Missense | - | 3.23E-05 |  | 0.034 | 0.934 | 1 | 14.41 |
| *TTN* | NM_001267550 | 49 | c.14141G>A | p.G4714D | rs750125429 | Missense | Yes | 3.23E-05 |  | 0.043 | 0.997 | 0.907 | 22.6 |
| *VCL* | NM_003373 | 6 | c.632delT | p.I211fs | - | Frameshift | - | - |  | - | - | - | - |
